# Using the posterior distribution of deviance to measure evidence of association for rare susceptibility variants

**DOI:** 10.1186/1753-6561-5-S9-S38

**Published:** 2011-11-29

**Authors:** Justo Lorenzo-Bermejo, Lars Beckmann, Jenny Chang-Claude, Christine Fischer

**Affiliations:** 1 Institute of Medical Biometry and Informatics, University Hospital Heidelberg, INF 305, 69120 Heidelberg, Germany; 2 Division of Cancer Epidemiology, German Cancer Research Centre, INF 280, 69120 Heidelberg, Germany; 3 Institute of Human Genetics, University of Heidelberg, INF 366, 69120 Heidelberg, Germany

## Abstract

Aitkin recently proposed an integrated Bayesian/likelihood approach that he claims is general and simple. We have applied this method, which does not rely on informative prior probabilities or large-sample results, to investigate the evidence of association between disease and the 16 variants in the *KDR* gene provided by Genetic Analysis Workshop 17. Based on the likelihood of logistic regression models and considering noninformative uniform prior probabilities on the coefficients of the explanatory variables, we used a random walk Metropolis algorithm to simulate the distributions of deviance and deviance difference. The distribution of probability values and the distribution of the proportions of positive deviance differences showed different locations, but the direction of the shift depended on the genetic factor. For the variant with the highest minor allele frequency and for any rare variant, standard logistic regression showed a higher power than the novel approach. For the two variants with the strongest effects on Q1 under a type I error rate of 1%, the integrated approach showed a higher power than standard logistic regression. The advantages and limitations of the integrated Bayesian/likelihood approach should be investigated using additional regions and considering alternative regression models and collapsing methods.

## Background

Association studies are substantially contributing to our knowledge of the genetic basis of human disease, but for most complex diseases the proportion of explained heritability remains at best modest [1]. Large variants, epistatic and parent-of-origin effects, additional layers of genetic variation (microRNA, DNA methylation, histone modification), and rare variants probably account for the missing heritability [2,3]. In this paper we explore the properties of a novel method of inference to quantify the evidence of association between a response variable (an indicator of disease status, the disease being common) and several independent variables, including the carriage of a rare variant.

Likelihood plays a central role in Bayesian, frequentist, and likelihood theory as a measure of strength of evidence. Based on the posterior distribution of the likelihood first described by Dempster, Aitkin has recently proposed an integrated Bayesian/likelihood approach that he claims is general and simple [4,5]. Bayes factors are usually applied to compare different statistical models and parameter values. Under the novel approach, likelihood ratios between models and parameter values are interpreted by using the full posterior distribution of the likelihood. A particular advantage of the method is that it does not rely on informative prior probabilities or large-sample results, and therefore it may be particularly suitable to identify rare susceptibility variants. We have applied this novel approach to investigate the evidence of association between disease and the 16 variants in the *KDR* gene provided by Genetic Analysis Workshop 17 (GAW17).

## Methods

Analyses were performed with knowledge of the underlying simulating model. Details on data simulation are provided by Almasy et al. [6]. In short, some *KDR* variants influence a quantitative risk factor Q1 with larger effects among smokers. Q1 correlates with a quantitative risk factor Q2 and with a normally distributed latent liability trait that increases with age and is higher in smokers. The dependent variable investigated in the present contribution (*y* = disease affection status) is a function of Q1, Q2, the liability trait, and additional risk factors.

We consider a baseline logistic regression model (model 1), where *y* depends on age (continuous variable), smoking status (dichotomous), and ethnicity (five categories). Model 2 includes age, smoking status, ethnicity, and a single genetic factor. Table 1 shows the list of investigated genetic factors. We consider individual variants 1 to 16, modeled by minor allele counts (additive model) and a collapsed genotype, and the presence of any rare variant, “rare” being defined as having a minor allele frequency less than 1%.

**Table 1:** Characteristics of the investigated variants in the *KDR* gene and results for the first replicate

Marker	Order	Position (MB)	Variant	Effect on Q1	MAF (%)	*P*-value	Deviance difference			
	
							Median	95% credible interval		Proportion > 0
C4S1859	1	55650982	T	–	0.14	0.18	−7.23	−17.66	2.75	0.06
C4S1861	2	55655860	T	0.56	0.22	0.30	−0.72	−11.53	10.04	0.44
C4S1868	3	55657190	G	–	0.07	0.05	−8.12	−18.62	−0.19	0.02
C4S1872	4	55665610	T	–	0.07	0.42	−1.46	−11.99	7.79	0.37
C4S1873	5	55665650	A	0.58	0.07	0.08	−7.65	−18.23	0.28	0.03
C4S1874	6	55665716	C	0.47	0.07	0.57	−1.03	−11.85	7.84	0.41
C4S1877	7	55667703	G	1.08	0.07	0.71	−0.99	−11.83	7.79	0.42
C4S1878	8	55667731	A	0.14	16.50	0.30	−0.09	−11.36	10.67	0.49
C4S1879	9	55668736	A	0.62	0.07	0.70	−0.99	−11.70	7.94	0.41
C4S1881	10	55671466	G	–	0.14	0.18	−7.26	−17.55	2.78	0.06
C4S1884	11	55674315	T	0.30	2.08	0.11	−2.30	−13.10	8.91	0.32
C4S1887	12	55675804	T	0.30	0.07	0.25	−2.09	−12.84	6.68	0.30
C4S1889	13	55676245	G	0.94	0.07	0.71	−0.86	−11.70	7.67	0.42
C4S1890	14	55676288	T	0.42	0.22	0.24	−1.39	−12.26	10.32	0.38
C4S1892	15	55679646	A	–	0.07	0.07	−7.71	−17.89	0.31	0.03
C4S1893	16	55679682	G	–	0.07	0.02	−9.77	−20.28	−1.95	0.01
Any rare variant						0.06	−3.14	−14.08	8.11	0.27

MAF, minor allele frequency. *P*-value is the probability value from standard logistic regression. “Any rare variant”: 17 out of 697 individuals carried at least one minor variant other than variant 8 and variant 11.

Under the two models, *y_i_ ~* Bernoulli (*π_i_*) with:(1)

where **x***_i_* contains the elements of the corresponding design matrices and **β** contains the model parameters (age, smoking status, ethnicity, and, for model 2, the single genetic factor). We consider noninformative, improper uniform prior probabilities for **β**. As a consequence, the joint posterior distributions of **β** are proportional to the likelihoods from the two logistic regression models, that is,(2)

with fixed *N* = 697 persons in the present exercise. We use a random walk Metropolis algorithm to make 10,000 draws from the posterior distribution under model 1 and 10,000 independent draws under model 2. The deviance is defined as minus twice the logarithm of the likelihood. Random draws from posterior distributions are used to simulate the distributions of deviance and deviance difference.

Power calculations rely on the probability values and on the proportions of positive deviance differences for the 200 replicates in the GAW17 data. To compare standard logistic regression with the novel approach, we use Wilcoxon rank sum tests on the null location shift and two-sample tests for the equality of proportions. Three different thresholds, 0.1, 0.05, and 0.01, are considered for probability values and proportions of positive deviance differences. All calculations and graphics are implemented with the free software environment R.

## Results

Table 1 shows some characteristics of the investigated variants. Effect sizes are shown for nonsmokers; they were 50% higher in smokers. Out of 16 variants, 10 variants influenced Q1, with variants 7 and 13 having the strongest effects (1.08 and 0.94, respectively). Minor allele frequencies (MAFs) varied from 0.1% to 17%. Out of 697 individuals, 17 (2.4%) carried at least one rare variant.

Detailed results for the first replicate from standard logistic regression and from the novel approach are also presented in Table 1. The probability value for a genetic effect based on a standard logistic regression model that also included age, smoking status, and ethnicity was 0.02 for variant 16 and 0.06 for “any rare variant.” The five probability values less than 0.10 were found for variants without a direct effect on Q1, with the exception of variant 5. The two variants with the strongest effects on Q1 (variants 7 and 13) showed the highest probability values (0.71 for both).

**Figure 1:**
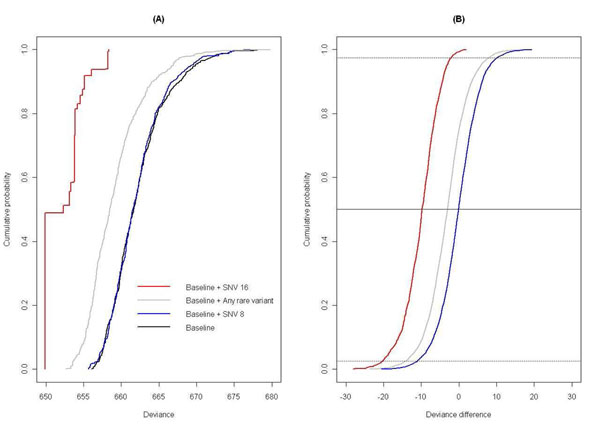
**Posterior cumulative distributions of (A) deviance and (B) deviance difference under four logistic regression models.**A baseline model that includes age, smoking status, and ethnicity was used as a reference to calculate deviance differences.

Figure 1A shows the posterior cumulative distributions of deviance for four logistic regression models. The lower the deviance (curves to the left), the higher the likelihood and the better the model. For each model, the lowest value of the deviance corresponds to the frequentist maximum likelihood. For example, the maximum likelihood was similar under model 1 (black curve) and when variant 8 was included (blue curve). If we consider the complete deviance distribution, we observe that the model that included any rare variant (gray curve) was better than model 1 and that a model with variant 16 (red curve) clearly dominated over the other three models.

Figure 1B shows deviance differences between three models that include a genetic factor and the reference model 1. The median deviance difference for the model with variant 16 (red curve) was −9.77 and the central 95% credible interval of the deviance difference was (−20.28, −1.95). The deviance difference was positive in 125 out of 10,000 draws. This proportion (125/10,000 ≈ 0.01) measures how strongly the data support a genetic effect of variant 16. Medians, 95% credible intervals, and proportions of positive deviance differences using model 1 as a reference are shown in Table 1 for the first replicate. In agreement with probability values from standard logistic regression, the strongest evidence of association based on the proportion of positive deviance differences was found for variants 16, 3, 5, and 15. In contrast to a probability value of 0.06, the proportion of positive deviance differences for “any rare variant” was 0.27. The proportion of positive deviance differences for variants 7 and 13, with the largest effect on Q1, was 0.42.

**Figure 2:**
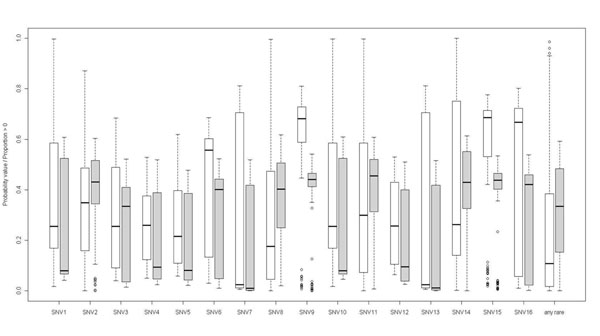
Box plots of probability values from standard logistic regression and proportions of positive deviance differences for the 200 replicates

Box plots of probability values and proportions of positive deviance differences in replicates 1 to 200 are shown in Figure 2. In general, probability values and proportions of positive deviance differences showed skewed distributions. The smallest median probability value and the smallest median proportion of positive deviance differences were observed for the two variants with the strongest effects on Q1 (variants 7 and 13).

Table 2 summarizes the probability values and proportions of positive deviance differences for the 200 replicates in the GAW17 data. Effects on Q1 and MAFs are also shown for convenience. For example, for variant 7 with an effect of 1.08 and MAF = 0.07%, the median probability value was 0.03 and 95% of the probability values belonged to the interval (0.01, 0.80). For this variant, the power of standard logistic regression was 70.5% (type I error rate, 10%), 65% (error rate, 5%) and 11.5% (error rate, 1%). The statistical power was identical for variant 13. Under standard logistic regression and a type I error rate of 10%, the power was 49% for “any rare variant” and it was 34.5% for variant 8, which had the highest MAF.

**Table 2:** Probability values, proportions of positive deviance differences, and power comparisons based on the 200 replicates

Variant	Effect on Q1	MAF (%)	Probability value						Positive deviance differences (%)						*P*_W_	*P*_0.1_	*P*_0.05_	*P*_0.01_
		
			Median	2.5th	97.5th	<0.1 (%)	<0.05 (%)	<0.01 (%)	Median	2.5th	97.5th	<0.1 (%)	<0.05 (%)	<0.01 (%)				
**1**			0.26	0.04	0.94	0.100	0.035	0.000	0.08	0.05	0.59	0.540	0.010	0.000				–
2	0.56	0.22	0.35	0.00	0.81	0.205	0.120	0.050	0.43	0.04	0.57	0.045	0.045	0.010	0.0002	<0.0001	0.01095	0.0402
3	–	0.07	0.25	0.05	0.57	0.280	0.015	0.000	0.34	0.02	0.48	0.480	0.315	0.000	<0.0001	<0.0001	<0.0001	–
4	–	0.07	0.26	0.08	0.49	0.100	0.000	0.000	0.09	0.03	0.47	0.510	0.280	0.000	<0.0001	<0.0001	<0.0001	–
5	0.58	0.07	0.22	0.07	0.50	0.180	0.000	0.000	0.08	0.03	0.45	0.615	0.350	0.000	<0.0001	<0.0001	<0.0001	–
6	0.47	0.07	0.56	0.04	0.66	0.200	0.155	0.000	0.40	0.01	0.49	0.330	0.255	0.000	<0.0001	0.00462	0.01861	–
7	1.08	0.07	0.03	0.01	0.80	0.705	0.650	0.115	0.01	0.00	0.50	0.690	0.690	0.485	<0.0001	0.82766	0.45667	<0.0001
8	0.14	16.50	0.18	0.00	0.95	0.345	0.255	0.095	0.40	0.06	0.58	0.065	0.020	0.000	<0.0001	<0.0001	<0.0001	<0.0001
9	0.62	0.07	0.68	0.03	0.79	0.060	0.040	0.005	0.44	0.02	0.51	0.055	0.055	0.020	<0.0001	1	0.63826	0.36808
10	–	0.14	0.26	0.04	0.94	0.100	0.035	0.000	0.08	0.05	0.59	0.540	0.010	0.000	<0.0001	<0.0001	0.17747	–
11	0.30	2.08	0.30	0.00	0.94	0.285	0.195	0.040	0.45	0.07	0.58	0.040	0.020	0.005	0.00106	<0.0001	<0.0001	0.04308
12	0.30	0.07	0.26	0.08	0.49	0.225	0.000	0.000	0.10	0.03	0.47	0.515	0.360	0.000	<0.0001	<0.0001	<0.0001	–
13	0.94	0.07	0.03	0.01	0.80	0.705	0.650	0.115	0.01	0.00	0.50	0.690	0.690	0.475	<0.0001	0.82766	0.45667	<0.0001
14	0.42	0.22	0.26	0.01	0.99	0.140	0.060	0.045	0.43	0.01	0.59	0.050	0.035	0.025	0.36355	0.00374	0.34708	0.41439
15	–	0.07	0.69	0.03	0.75	0.095	0.040	0.000	0.44	0.01	0.51	0.095	0.095	0.020	<0.0001	1	0.04627	0.13167
16	–	0.07	0.67	0.01	0.78	0.325	0.235	0.000	0.42	0.00	0.51	0.320	0.320	0.155	<0.0001	1	0.07399	<0.0001
Any rare variant			0.11	0.00	0.91	0.490	0.395	0.205	0.33	0.01	0.57	0.165	0.095	0.025	<0.0001	<0.0001	<0.0001	<0.0001

MAF is the minor allele frequency. 2.5th and 97.5th are percentiles. *P*_W_ is the probability value from a Wilcoxon rank sum test on the null location shift. *P*_0.1_ is the probability value from a two-sample test for equality of proportions (percentage of probability values smaller than 0.1 and percentage of proportions of deviance differences under 0.1); the same applies to *P*_0.05_ and *P*_0.01_.

Based on the integrated Bayesian/likelihood approach, the smallest median proportion of positive deviance differences (0.01) and the highest power (69% for a type I error rate of 10%) were also found for variants 7 and 13. Wilcoxon rank sum tests indicated that the distribution of probability values and the distribution of the proportions of positive deviance differences showed different locations, but the direction of the shift was variable (see Figure 2). For variant 8 and for “any rare variant,” standard logistic regression showed a higher power than the novel approach (probability values from Wilcoxon rank and equality of proportion tests < 0.0001). For variants 7 and 13 and a type I error rate of 1%, the integrated approach showed a higher power than standard logistic regression.

## Discussion and conclusions

Genome-wide association studies usually rely on the common disease/common variant hypothesis: Polymorphisms are genotyped and their association with disease is investigated. The identified, most often indirect associations are assumed to reflect a shared inheritance of the genotyped markers and linked causal variants [7]. The first aim of these studies is to detect association regions, mostly by relying on significance testing and summarizing results by probability values. When more precise hypotheses and more refined studies have been set up (e.g., focusing on a specific chromosomal region and incorporating genotypes from public data repositories), the subsequent goal is to quantify and model associations and to identify causal variants underlying the association signal.

The investigated genotypes are usually ranked according to plausibility of causal association. Ranks are most often based on probability values, although probability values have important disadvantages compared to Bayes factors [8]. Probability values are often misinterpreted as the probability of no association given the observed data, but they actually measure the probability of the data given no association. The ranking of markers based on probability values is difficult to interpret, because probability values depend on factors that influence power, such as allele frequency and sample size. In addition, Bayes factors may discriminate between causal variants and markers better than probability values. However, a limitation of Bayes factors is the need to define proper prior probabilities in order to compute an integrated likelihood. Aitkin recently proposed a Bayesian/likelihood approach that allows incorporation of a priori external information if desired, but it also allows use of improper noninformative prior probabilities. The relative simplicity and the independence on large-sample results are important advantages of the method. Limitations include computation time and the necessity to carefully check for convergence in Markov chain Monte Carlo methods. The novel approach could also be applied to investigate the role of additional layers of genetic variation (gene methylation and expression), once its potential over standard methods has been investigated.

The marked differences between the data in Table 1, which shows results for the first replicate, and Table 2, which summarizes results for all 200 replications, reflect the importance of external validation of initial association signals. For example, in the first replicate, the highest probability value and the highest proportion of positive deviance differences were observed for the two variants with the strongest effects on Q1 (variants 7 and 13). Table 2 shows that the average statistical power was highest for these two variants.

Our analyses have some limitations. We have considered one single gene and used a simplistic method to collapse rare variants; details on more sophisticated collapsing methods can be found in the overview by Dering et al. [9]. The baseline model was also fixed, and gene-smoking interactions were not investigated. The distribution of deviance differences was smooth, but the deviance distribution was discrete. Our reference method was standard logistic regression; a comparison with Bayesian methods relying on Bayes factors would also be of interest. Our goal with this analytical exercise was to gain experience with a novel technique that may offer some advantage to identify rare susceptibility variants.

## Competing interests

The authors declare that there are no competing interests.

## Authors’ contributions

JLB conceived of the study, performed statistical analyses and drafted the manuscript. CF contributed to the study design and helped to draft the manuscript. LB extracted the necessary data for the subsequent statistical analysis. All authors read, commented on and approved the final version of the manuscript.
